# Individual Differences in Hatching Time Predict Alcohol Response in Zebrafish

**DOI:** 10.3389/fnbeh.2019.00166

**Published:** 2019-07-23

**Authors:** Maria Elisa Leite-Ferreira, Heloysa Araujo-Silva, Ana Carolina Luchiari

**Affiliations:** Departamento de Fisiologia e Comportamento, Centro de Biociências, Universidade Federal do Rio Grande do Norte, Natal, Brazil

**Keywords:** ethanol, egg emergence, anxiety, locomotion, personality, *Danio rerio*

## Abstract

There are significant individual differences in response to alcohol: some people seem to exhibit higher alcohol sensitivity, while others are more resistant. These differences are related to alcohol metabolism, inherited traits, environmental/social pressure, personal habits and other indeterminate causes. In order to test how individual differences in hatching time are related to behavioral response to different alcohol concentrations, we separated zebrafish larvae into two categories according to egg emergence time: eggs hatched between 48 and 72 hours post-fertilization (hpf) were considered early emerging (EE), while those hatched from 72 to 96 hpf were considered late emerging (LE). On the 30th day post fertilization, EE and LE fish were exposed to four alcohol concentrations: 0.00% (control), 0.10%, 0.25% and 0.50%, and behavior was recorded for 60 min. We observed average and maximum swimming speed, distance traveled, and freezing time (immobility that indicates state of anxiety). For EE fish, 0.10% alcohol did not change behavior, while 0.25% and 0.50% increased freezing and decreased locomotion. By contrast, LE fish increased locomotion when exposed to both 0.10 and 0.25% alcohol, and increased freezing time at 0.50% alcohol. These results show that zebrafish behavioral profiles exhibit different sensitivities to alcohol, likely due to traits that can be tracked from early life stages and may indicate individuals’ predisposition to alcohol tolerance and dependence.

## Introduction

Alcohol use is an age-old problem. It is the most commonly abused drug and has a massive impact on society (WHO, 2018). The neurological effects of alcohol manifest themselves in short and long-term use, ranging from increased aggressiveness, loss of motor control and single-event memory failure (Roseribloom et al., 2004; Quoilin et al., 2013; Amorim et al., 2017) to a highly debilitating state such as Wernicke-Korsakoff syndrome (Sullivan and Pfefferbaum, 2009). However, there are enormous individual differences in response to alcohol, with some people exhibiting higher alcohol sensitivity, while others are more resistant (van Beek et al., 2014). These differences are related to alcohol metabolism, inherited traits, environmental/social pressure, personal habits and other indeterminate causes (Bartholow et al., 2003; Buisman-Pijlman et al., 2014; Gullo and Potenza, 2014). Screening individual differences in order to detect traits associated with alcohol abuse are both difficult and expensive, but some profiles seem to indicate a greater likelihood of developing alcoholism and merit more thorough investigation (Cotton, 1979; Roman and Colombo, 2009; Araujo-Silva et al., 2018).

Individual variations in phenotype and behavior were long considered noise rather than the result of several different biological processes. However, in recent decades, the study of trait variability among individuals of the same species, so-called personality, temperament, or copying strategies (Gosling and John, 1999; Koolhaas et al., 1999; Réale et al., 2007) has been gaining ground since individuals exhibit different responses to similar environmental challenges (Gosling and John, 1999; Gosling et al., 2003), which reflect both genetic and environmental determinants. For instance, it is known that individuals differ in their metabolic rate (Braga Goncalves et al., 2015; Auer et al., 2018; Pettersen et al., 2018), vulnerability to disease (Cavigelli, 2005; MacKenzie et al., 2009), cortisol response to stress (Overli et al., 2002; Øverli et al., 2006, 2007; Frost et al., 2007; Kristiansen and Fernö, 2007; Silva et al., 2010) and more recently, a number of specific gene transcripts in the brain (MacKenzie et al., 2009) in addition to the response to drugs of abuse such as alcohol (Araujo-Silva et al., 2018).

In studies investigating individual differences in several species, including mammals, birds, and fish (Baugh et al., 2017; Araujo-Silva et al., 2018; Ferreira et al., 2018), a common idea is that the behavioral profile is usually accompanied by physiological responses. Several authors suggest that active vs. passive individuals can be characterized at either end of a continuum, with many intermediate profiles in between. These two extreme profiles, defined by Koolhaas et al. (1999) as “proactive” and “reactive,” display opposite physiological and behavioral responses, such as sympathethic and parasympathetic reactivity (Verbeek et al., 2008), testosterone levels (Koolhaas et al., 1999), basal cortisol and hypothalamus-pituitary-interrenal (HPI) axis activity (Koolhaas et al., 1999; Øverli et al., 2005; Silva et al., 2010), reactivity to escape stressors (Silva et al., 2010), feeding motivation, exploration and risk-taking in novel environments (Øverli et al., 2006, 2007; Frost et al., 2007; MacKenzie et al., 2009), and aggressiveness (Øverli et al., 2004). As such, it seems that the concept of individual differences refers to interindividual variation in energy consumption to cope with situations throughout life; thus, the metabolic rate of the two most contrasting profiles should be markedly different.

Given that they appear very early in life, it has been reported that individual differences can be tracked from the spawning nest in salmonids, which may be linked to some behavioral and physiological characteristics in juvenile and adult fish (Vaz-Serrano et al., 2011; Andersson et al., 2013; Thörnqvist et al., 2015; Rosengren et al., 2017). While increased vulnerability to predation is an obvious disadvantage in early hatching time, benefits include increased access to territory and food (Brännäs, 1995). Screening behavior and physiological responses in early and late emerging fish suggest similarities with the proactive and reactive profiles: early emerging rainbow trout and Atlantic salmon were shown to be bolder, dominant and with lower brain serotonin levels during stress, resembling the proactive profile, while the opposite was observed for the late emerging fish (Metcalfe and Thorpe, 1992; Vaz-Serrano et al., 2011; Andersson et al., 2013; Thörnqvist et al., 2015). This evidence suggests that the timing of emergence is linked to boldness and proactive style, another trait that may indicate individual profile.

In recent years, the use of zebrafish (*Danio rerio*) as an animal research model to evaluate the effect of alcohol on behavior has increased (Gerlai et al., 2000; Irons et al., 2010; Tran and Gerlai, 2013; Chacon and Luchiari, 2014; Luchiari et al., 2015). Their high genetic and physiological similarity with humans enables translational research (Araujo-Silva et al., 2018). Furthermore, characteristics such as external fertilization and the transparency of the fish in the embryonic and larval stages make it possible to study the developing nervous system (Irons et al., 2010). Embryonic development is fast, whereby an ovule that has been fertilized develops into a larva with a heartbeat and eyes in 24–48 h (Kimmel et al., 1995), and a rich behavioral repertoire within a few days (Budick and O’Malley, 2000; Colwill and Creton, 2011). The zebrafish response to alcohol has been shown to resemble that of humans, making them a feasible model for the study of alcoholism, its variations and consequences (Gerlai et al., 2000). Thus, considering the differences between individuals in a population and that these differences may affect how they respond to a psychotropic drug as alcohol, we aimed at evaluating the effects of different alcohol concentrations (0.00%, 0.10%, 0.25% or 0.50%) on early and late emerging profiles in zebrafish (*Danio rerio*). Our hypothesis was that individual differences observed in early stages of development affect the way animals respond to alcohol later in life.

## Materials and Methods

### Animal Housing, Maintenance and Breeding

Adult zebrafish (*Danio rerio*, wild-type, both sexes) were obtained from a local farm (Natal, Brazil) and held in 50 L tanks with a multistage filtration system at the fish vivarium of the Federal University of Rio Grande do Norte (UFRN). Temperature, pH, and oxygen were maintained at 28°C, 6.7 and 6 mg/L, respectively. A 12 h light-dark cycle was adopted. Fish were fed twice a day with commercial pelleted food (Alcon Basic^®^, 45% protein; 5% fat, Alcon, Brazil) and live brine shrimp (Premium grade, Brine Shrimp Direct, Ogden, UT, USA).

Every other day, two female and three male zebrafish from the stock were placed in breeding tanks (30 × 15 × 20 cm) filled with 3 L of system water. An acrylic plate full of small holes was placed on the bottom of the tank to prevent the fish from accessing the eggs. Plastic plants were used to enrich the tank and promote breeding. The breeding group were placed in the tanks at around 5 pm and kept in the same room conditions as the stock (28°C, 12L:12D). Fertilization was performed by natural spawning, which usually occurred during the first hour of daylight.

A total of 50 breeding groups (100 females and 150 males) were used to obtain the total number of eggs for this study. The eggs were collected, counted and transferred to Petri dishes, which were placed in an incubator at 28°C and checked daily for mortality and hatching time. Eggs were observed from 24 to 96 hours post-fertilization (hpf), every 2 h to remove hatched fry, which were placed in a separate Petri dish. Eggs hatched between 48 and 72 hpf were considered “early emerging” (EE), while those hatched from 72 to 96 hpf were denominated “late emerging” (LE). EE and LE larvae were held in separated Petri dishes kept in the incubator until 120 hpf. Next, the larvae were transferred to plastic trays (BioPrátika 30.3 × 22.1 × 7.5 cm; filled with 1 L of system water) and exogenous feeding initiated. The larvae were fed powdered food dissolved in system water (Alcon Alevinos^®^, 44% protein; 5% fat, Alcon, Brazil) three times a day until 12 days post-fertilization (dpf). From 12 dpf on, the larvae were fed powdered food and brine shrimp until 30 dpf. A silicon tube connected to an air pump supplied oxygen to the water in the trays. At 15 dpf, fish were removed to tanks with water recirculating system where the volume was increased to 2 L and debris were washed away continuously. All the procedures were approved by the Animal Ethics Committee of UFRN (CEUA 122.055/2018).

### Experimental Design and Alcohol Exposure

At 30 dpf, EE and LE larvae were divided into eight groups and exposed to four alcohol concentrations: 0.00% (control), 0.10%, 0.25% and 0.50%. Thus, different alcohol concentrations and hatching profiles could be tested from the groups formed (four EE groups and four LE groups): EE 0.00% alcohol (*n* = 13), LE 0.00% alcohol (*n* = 13), EE 0.10% alcohol (*n* = 13), LE 0.10% alcohol (*n* = 13), EE 0.25% alcohol (*n* = 13), LE 0.25% alcohol (*n* = 13), EE 0.50% alcohol (*n* = 13), and LE 0.50% alcohol (*n* = 13). For alcohol exposure, 99% absolute ethanol (Dinâmica, Química contemporânea Ltd, Brazil) was diluted into the system water to achieve the three concentrations used (0.10%, 0.25% and 0.50%).

Cell culture plates containing six wells were used for behavioral screening. The solution containing alcohol (0.00, 0.10, 0.25 or 0.50%) was used to fill the wells and fish was individually transferred to the well (one fish per well). Fish behavior was recorded from above for 60 min using a digital camera (Sony DCR-SX45 Digital Video Camera Recorder). The video files were transferred and analyzed using a video tracking program developed at MatLab (Pinheiro-da-Silva et al., 2017). Behavior was evaluated every min for 60 min, creating time course screening of the hatching profile of fish exposed to each alcohol concentration. The following parameters were quantified: average and maximum swimming speed, total distance traveled, and time spent immobile (freezing).

### Statistical Analysis

Behavioral data were assessed to check for outliers, homogeneity, normality, zero trouble, collinearity and independent variables, as suggested by Zuur et al. (2010) Since our data were longitudinal (every minute for 60 min, obtaining repeated measures of the same animal), we used mixed effects modeling to develop a model for the response variable (each behavioral parameter evaluated) and explanatory variable (profile: EE or LE, alcohol concentration: 0.00, 0.10, 0.25, 0.50%, and time: 60 min). The mixed model used showed random effect factors due to the behavioral variation within the groups, fixed effect factors caused by the alcohol concentration effects observed, and standard error.

We used the glmmPQL command from the MASS package (Venables and Ripley, 2003) of the R program (Team, 2015) to develop the mixed model. The response variable freezing varied between 0 and 60 s, with a binomial distribution error and logit link function (according to Zuur et al., 2010). The response variables average speed, maximum speed and distance traveled were positive continuous quantitative data, not including zero (*Y* > 0); thus, a goodness-of-fit test was performed to determine the best distribution function. The gamma distribution function best fit these variable data (link function = inverse). In all cases, the *post hoc* comparisons between treatments of each model were made using Tukey’s test in the “lsmeans” package (Lenth and Hervé, 2015). The significance level was set at *p* < 0.05.

Following the time-course analysis, we conducted a two-way analysis of variance (ANOVA) test to evaluate the main effect of alcohol concentration (four levels: 0.00, 0.10, 0.25, and 0.50%) and the emerging profile (two levels: early and late emerging) during the last 20 min of alcohol exposure, as well as the interaction between alcohol concentration and profile. Thus, the first response to the novel environment could be ignored and fish behavior under the influence of alcohol highlighted. When ANOVA exhibited statistical significance, we used Tukey’s HSD *post hoc* test. The significance level was set at 0.05.

## Results

**Figure 1:**
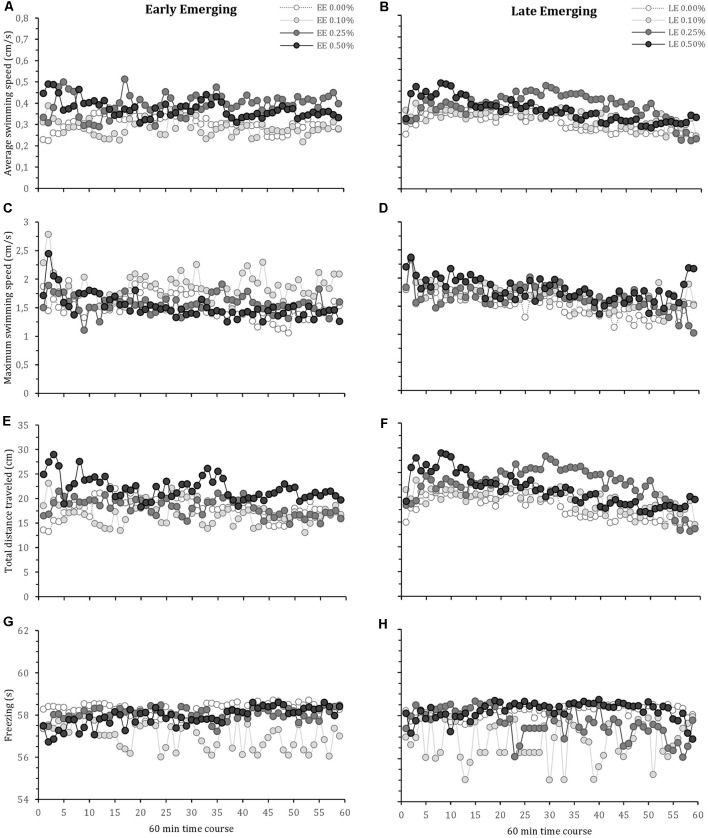
Time-course behavioral changes during 60-min alcohol exposure in early and late emerging zebrafish. Early and Late emerging profiles were determined by the emergence time form the egg. Eggs hatched up to 72 hours post-fertilization (hpf) were considered early emerging (EE) and eggs hatched after 72 hpf were the late emerging (LE) fish. EE and LE larvae were kept up to 30 days old and then were exposed to alcohol concentrations of 0.00% (control), 0.10%, 0.25% or 0.50% for 60 min during which behavior was recoded. Graphs **(A)** and **(B)** show EE and LE fish average swimming speed, respectively. Graphs **(C)** and **(D)** present EE and LE fish maximum swimming speed, respectively. Graphs **(E)** and **(F)** are total distance traveled for EE and LE fish, respectively. Graphs **(G)** and **(H)** depict freezing time for EE and LE fish, respectively. Sample sizes (n) were 13 for each group. Mean are shown for every 1-min intervals of the total 60 min recording.

**Figure 2:**
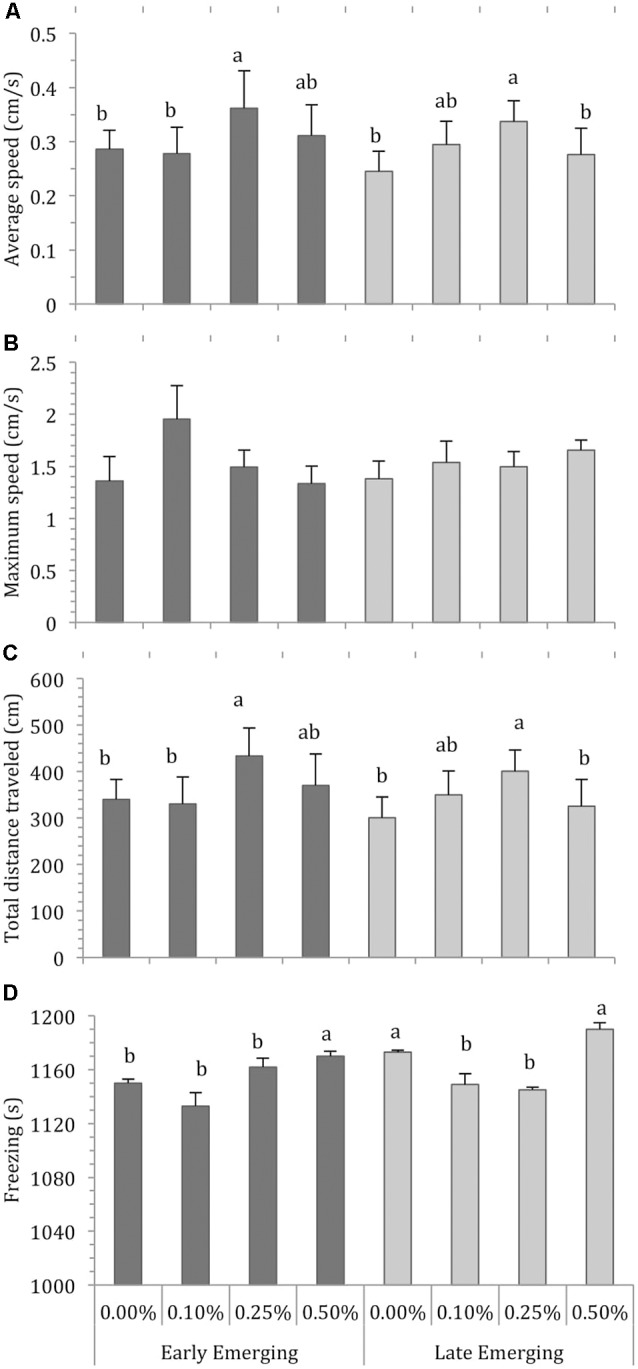
Comparison of Early Emerging and Late Emerging zebrafish following alcohol exposure. Fish emerging profiles were determined according to eggs hatching time: individuals that hatched up to 72 hours post-fertilization (hpf) were the early emerging fish and those that hatched after 72 hpf were the late emerging fish. Early and late fish were exposed to alcohol concentration (0.00%, 0.10%, 0.25% or 0.50%) at 30 days post fertilization and behavior was recoded. Sample sizes (n) were 13 for each group. Bars indicates zebrafish locomotor behavior [mean + standard error of the mean (SEM)] during the last 20 min of 60-min alcohol exposure period: **(A)** average swimming speed, **(B)** maximum swimming speed, **(C)** total distance traveled, and **(D)** freezing. Different letters indicate statistical differences between groups analysis of variance (ANOVA, *p* < 0.05).

The locomotor parameters of the fish hatching profile exposed to each alcohol concentration for 60 min are presented in Figure 1. Figure 2 depicts average locomotor response during the last 20 min of alcohol exposure.

Mixed model comparison showed that average zebrafish swimming speed during acute alcohol exposure was significantly changed due to fish profile (GLMM, *χ*^2^ = 5.32, *df* = 1, *p* = 0.02; Figures 1A,B) and alcohol concentration (GLMM, *χ*^2^ = 37.38, *df* = 3, *p* < 0.001; Figures 1A,B), but did not change over time (GLMM, *χ*^2^ = 3.14, *df* = 1, *p* = 0.07; Figures 1A,B). The interactions terms that showed statistical significance were profile vs. treatment (GLMM, *χ*^2^ = 4.54, *df* = 3, *p* = 0.05), profile vs. time (GLMM, *χ*^2^ = 8.44, *df* = 1, *p* = 0.003), and alcohol concentration vs. time (GLMM, *χ*^2^ = 19.64, *df* = 3, *p* < 0.001). The *post hoc* comparison test (lsmeans) between groups indicates that EE 0.01% showed the lowest average swimming speed, significantly different from EE 0.25%, EE 0.50%, LE 0.10%, LE 0.25% and LE 0.50%. Lsmeans also demonstrated that the highest average speed was obtained by LE 0.25%, significantly different from the other groups.

For maximum swimming speed, mixed model comparison indicated statistical significance due to alcohol concentration (GLMM, *χ*^2^ = 12.17, *df* = 3, *p* = 0.007; Figures 1C,D) and over time (GLMM, *χ*^2^ = 14.37, *df* = 1, *p* < 0.001; Figures 1C,D), but no change with respect to fish profile (GLMM, *χ*^2^ = 0.41, *df* = 1, *p* = 0.52; Figures 1C,D). The interaction terms that displayed statistical significance were alcohol concentration vs. time (GLMM, *χ*^2^ = 27.19, *df* = 3, *p* < 0.001), while the other interactions were not significant (profile vs. alcohol concentration: GLMM, *χ*^2^ = 7.37, *df* = 3, *p* = 0.06; profile vs. time: GLMM, *χ*^2^ = 0.81, *df* = 1, *p* = 0.37). The *post hoc* comparison test (lsmeans) showed that EE 0.10% obtained the highest maximum speed, followed by LE 0.50%, while the control groups (EE 0.00% and LE 0.00%) exhibited the lowest maximum speed (Figure 2B).

The mixed model comparison of total distance traveled showed statistical significance for fish profile (GLMM, *χ*^2^ = 5.16, *df* = 1, *p* = 0.02; Figures 1E,F) and alcohol concentration (GLMM, *χ*^2^ = 39.61, *df* = 3, *p* < 0.001; Figures 1E,F), but there was no change over time (GLMM, *χ*^2^ = 1.21, *df* = 1, *p* = 0.27; Figures 1E,F). The interaction terms showed statistical significance for profile vs. treatment (GLMM, *χ*^2^ = 13.46, *df* = 3, *p* = 0.003), profile vs. time (GLMM, *χ*^2^ = 7.82, *df* = 1, *p* = 0.005), and alcohol concentration vs. time (GLMM, *χ*^2^ = 12.62, *df* = 3, *p* = 0.005). Lsmeans *post hoc* test showed that EE 0.010% and LE 0.25% traveled the shortest and longest distance, respectively.

Freezing behavior, which reflects a state of immobility related to fear and anxiety usually observed when zebrafish are placed in a novel environment, is presented in Figure 1D. A mixed model comparison reveals statistical significance in fish profile (GLMM, *χ*^2^ = 10.41, *df* = 1, *p* = 0.003; Figures 1G,H), alcohol concentration (GLMM, *χ*^2^ = 3.03, *df* = 3, *p* < 0.001; Figures 1G,H) and over time (GLMM, *χ*^2^ = 4.75, *df* = 1, *p* = 0.03; Figures 1G,H). Alcohol concentration vs. time showed statistical significance (GLMM, *χ*^2^ = 27.19, *df* = 3, *p* < 0.001), the interactions between profile vs. alcohol concentration (GLMM, *χ*^2^ = 3.37, *df* = 3, *p* = 0.006) and profile vs. time (GLMM, *χ*^2^ = 3.81, *df* = 1, *p* = 0.03) were also was statistical significant. The *post hoc* comparison test (lsmeans) showed that EE 0.10% and LE 0.10% obtained the lowest freezing values, followed by LE 0.25%, while the other groups obtained the highest freezing values.

Depending on their profile, fish display the effects of alcohol concentration during the last 20 min of exposure because they are already habituated to the new environment and display no novelty-related anxiety. Figure 2 presents the average values of the locomotor parameters measured during the last 20 min of alcohol exposure in fish from the early and late emerging profiles. Two-way ANOVA of the average swimming speed data revealed a significant effect of alcohol exposure (*F*_(3,103)_ = 4.09, *p* = 0.008). The main effect of emergence profile was non-significant (*F*_(1,103)_ = 0.75, *p* = 0.39). The profile vs. alcohol concentration interaction term was non-significant (*F*_(3,103)_ = 0.29, *p* = 0.83). Tukey’s HSD test showed that EE 0.25% and LE 0.25% differed significantly (*p* < 0.05) from the other groups and LE 0.10% was significantly different from LE 0.00% (Figure 2A).

For maximum speed, two-way ANOVA found that the main effect of alcohol concentration was non-significant (*F*_(3,103)_ = 0.86, *p* = 0.46), as were the effects of profile (*F*_(1,103)_ = 3.23, *p* = 0.07) and interaction terms (*F*_(3,103)_ = 0.78, *p* = 0.50; Figure 2B). Two-way ANOVA analysis for total distance traveled showed a significant effect of alcohol concentration (*F*_(3,103)_ = 4.58, *p* = 0.01), but the effects of profile (*F*_(1,103)_ = 0.74, *p* = 0.38) and profile vs. alcohol were non-significant (*F*_(3,103)_ = 0.29, *p* = 0.82). Tukey’s HSD test indicated that EE 0.25%, LE 0.10% and LE 0.25% differed significantly (*p* < 0.05) from the other groups (Figure 2C). Finally, analysis of freezing data showed that the effect of both alcohol exposure (*F*_(3,103)_ = 27.94, *p* < 0.01) and emergence profile (*F*_(1,103)_ = 35.52, *p* < 0.01) were significant. The interaction terms profile vs. alcohol concentation were significant (*F*_(3,103)_ = 5.03, *p* < = 0.002). Tukey’s HSD test indicated that EE 0.50%, LE 0.00% and LE 0.50% differed significantly (*p* < 0.05) from the other groups (Figure 2D).

## Discussion

In this study, we showed the effects of alcohol exposure on different zebrafish profiles according to fry emerging time: early emerging (EE) and late emerging (LE). Early emerging (EE) zebrafish showed lower anxiety-like behavior compared to their late emerging (LE) counterparts, suggesting that some behavioral reactions may be established very early in life. Moreover, these profiles responded differently to alcohol exposure, a psychoactive drug that alters brain biochemistry and ultimately reflects on animal behavior. EE zebrafish were affected by 0.25% and 0.50% alcohol, showing increased and decreased locomotion repectively but 0.10% alcohol did not change EE fish behavior. However, LE animals increased swimming and decreased freezing when exposed to 0.10% and 0.25% alcohol, while 0.50% alcohol caused increased freezing response. These results indicate that both profiles are affected by alcohol, but very low concentrations such as 0.10% are enough to cause behavioral changes in late emerging fish, while still tolerated by EE individuals, suggesting that different sensitivity to alcohol can be tracked from an early stage.

Individual differences in behavioral profile are molded by evolution (Sih et al., 2004; Colléter and Brown, 2011), thereby exerting strong genetic influence. In a natural environment, emergence from the spawning nest is a critical ontogentetic shift subjected to high selection pressure. From this moment on, individual experiences largely affect behavior and may have consequences for fitness (Dingemanse et al., 2004; Brown et al., 2007a,b). Several studies correlate emergence time from the egg with personality traits, including exploration, risk-taking, aggressiveness, and metabolic rate (Biro and Stamps, 2008). Early emerging fry usually require less time to overcome stress (Killen et al., 2011; Vaz-Serrano et al., 2011) higher aggression (Lahti et al., 2002; Killen et al., 2012), dominance behavior and boldness, resembling the proactive copying style (Martins et al., 2011; Vaz-Serrano et al., 2011; Andersson et al., 2013). For example, Metcalfe and Thorpe (1992) and Metcalfe et al. (1995) showed that early emerging Atlantic salmon are socially dominant, exhibit a higher metabolic rate and reach smoltification earlier than late emerging fry, while Rosengren et al. (2017) suggested higher risk behavior during stress in early emerging fish. Our results for EE and LE zebrafish exposed to 0.0% alcohol (control) show that the former are less sensible to environmental changes, displaying lower freezing behavior than LE. Zebrafish are naturally explorative, but usually increase fear/anxiety response (i.e., freezing) in novel places (Wong et al., 2010; Jesuthasan, 2012; Stewart et al., 2012), which is an adaptive behavior since unknown sites may pose unforeseen threats. However, the fear/anxiety response is expected to decrease over time, but EE and LE fish took different time periods to adjust to the new tank, which seems to be related to their profile. According to Vaz-Serrano et al. (2011) and Andersson et al. (2013), there is a correlation between emergence time and stress coping styles, the early and late emerging fish corresponding to the proactive and reactive profile, respectively. As such, LE zebrafish should take longer to reduce anxiety in a novel environment.

However, alcohol exposure showed potential to change this scenario. Alcohol is a biphasic drug that initially causes an anxiolytic effect, making one more explorative, less fearful and more likely to take risks (Addicott et al., 2007; Irons et al., 2010; Araujo-Silva et al., 2018). On the other hand, increasing alcohol concentration has the opposite effect: it heightens anxiety, decreases exploration and leads to a depressive state (Charness et al., 1989; Koike and Sobue, 2006; Campbeel et al., 2013; Amorim et al., 2017; Araujo-Silva et al., 2018). Very low alcohol concentrations are not expected to change behavior. A low dose is considered subclinical, that is, it causes slight neurochemical changes in brain function without affecting behavior (Careau et al., 2008). However, the low concentration used in the present study (0.10% alcohol) which did not alter EE response, led to decreased anxiety-like behavior and increased locomotion in LE fish. Not only did the very low concentration affect LE behavior, but the other alcohol concentrations also induced locomotor alterations.

Differences between the two profiles (EE vs. LE) may explain why they showed different responses to alcohol exposure. One possibility is the metabolic level exhibited by these profiles. Several studies have related early emergence to increased metabolic rate in fish (Vaz-Serrano et al., 2011; Andersson et al., 2013; Braga Goncalves et al., 2015; Rosengren et al., 2017; Auer et al., 2018; Pettersen et al., 2018), which suggests that EE fish may metabolize alcohol faster than LE, decreasing blood levels and accelerating drug excretion earlier than LE fish. This hypothesis seems plausible, since only the higher alcohol concentrations (0.25 and 0.50%) affected EE, while 0.10, 0.25 and 0.50% alcohol altered LE behavior, leading to the conclusion that slower alcohol processing allowed the lower concentration to enter the brain and affect behavior expression.

Although Careau et al. (2008) explicitly correlated metabolic traits to different personalities, more than only metabolism level is needed to explain why EE fish became less anxious when exposed to 0.25 and more anxious with 0.50% alcohol, while their LE counterparts reduced anxiety-like behavior under 0.10 and 0.25% and increased this response with 0.50% alcohol. Thus, we believe that the neurotransmission underlying individual differences may have affected the alcohol outcomes.

Acute alcohol exposure undoubtedly acts on several brain neurotransmitter systems, promoting behavioral modifications that depend on the amount of alcohol available and neural activity level. For instance, a small amount of alcohol is considered anxiolytic (Tran et al., 2016), since it inhibits the glutamatergic system and stimulates the GABAergic system (Rico et al., 2011) causing an initial relaxation effect. Additionally, alcohol activates serotonin and dopamine release, two important neurotransmitters related to arousal states (i.e., locomotor activity and responsiveness; Chiu and Prober, 2013) and anxiety behavior (Kalueff et al., 2007; Banerjee, 2014), respectively. This response was observed in LE zebrafish exposed to both 0.10 and 0.25% alcohol, given that they reduced freezing behavior and increased exploration, corroborating other authors who reported the anxiolytic effects of lower alcohol doses and anxiogenic effects of higher doses (Gerlai et al., 2000; Mathur and Guo, 2011; Amorim et al., 2017).

In fact, the neurotransmitter systems have been suggested to differ between individual personalities. Lower serotonin and higher dopamine levels characterize proactive and bolder individuals, while reactive and shyer animals exhibit higher serotonin and lower dopamine levels (Koolhaas et al., 1999, 2010; Silva et al., 2010; Backström and Winberg, 2017). Thus, when alcohol interacts with serotonergic and dopaminergic brain activity, the outcomes of bold and shy animals are expected to be complete opposites. For instance, alcohol increases dopamine transmission, which is suggested to be related to the user’s initial feeling of pleasure, and likely much more intense in shy than bold animals, due to the lower dopamine levels in the former. In this respect, we believe that alcohol is a psychoactive drug, perceived differently by each profile, making shyer individuals more prone to developing dependence than their bold counterparts.

In the present study, EE and LE zebrafish showed different responses to alcohol. Our results suggest that 0.10 and 0.25% alcohol decreased anxiety-like behavior in LE zebrafish, while 0.50% alcohol increased anxiety. By contrast, 0.10% alcohol did not change EE behavior, but 0.25% alcohol exerted anxiolytic effects and 0.50% induced an anxiogenic response. Thus, alcohol responsiveness may be related to the intrinsic characteristics of individuals, including genetic predisposition, metabolic rate and neurotransmitter level in the brain. Alcohol, one of the most abused drugs in the world, is responsible for more than 3 million deaths a year (WHO, 2018), making prevention and effective treatments a daunting challenge. In this regard, using zebrafish to show that individual features observed since very early in life may be an influencing factor on alcohol responsiveness and dependence could lead to new studies on the mechanisms of susceptibility to alcoholism.

## Conclusion

This study is the first to show that early life traits may be related to alcohol responsiveness, and it is important to underscore that the environment has a significant impact on behavior and decision making. Thus, life experiences should also be considered an indicator of alcohol dependence. Furthermore, future research focusing on individual differences in levels of neurotransmitters such as dopamine and serotonin before and after alcohol exposure may help understand why some profiles show greater predisposition to alcoholism than others, in addition to suggesting prospective treatments. Knowledge on how different individuals cope with the environment in order to survive and succeed is essential to understanding vulnerability to diseases such as alcoholism, a condition that the zebrafish has been contributing to elucidate, reinforcing its value in translational research.

## Data Availability

The datasets generated for this study are available on request to the corresponding author.

## Ethics Statement

All the procedures were approved by the Animal Ethics Committee of the Federal University of Rio Grande do Norte (CEUA 122.055/2018).

## Author Contributions

ML-F and AL conceived and designed the experiments and wrote the article. ML-F and HA-S perfomed the experiments. HA-S analyzed the data.

## Conflict of Interest Statement

The authors declare that the research was conducted in the absence of any commercial or financial relationships that could be construed as a potential conflict of interest.
